# New Insights Into Wall Polysaccharide *O*-Acetylation

**DOI:** 10.3389/fpls.2018.01210

**Published:** 2018-08-21

**Authors:** Markus Pauly, Vicente Ramírez

**Affiliations:** Institute for Plant Cell Biology and Biotechnology – Cluster of Excellence on Plant Sciences, Heinrich Heine University Düsseldorf, Düsseldorf, Germany

**Keywords:** *O*-acetylation, cell wall, polysaccharides, biosynthesis, mechanism

## Abstract

The extracellular matrix of plants, algae, bacteria, fungi, and some archaea consist of a semipermeable composite containing polysaccharides. Many of these polysaccharides are *O*-acetylated imparting important physiochemical properties to the polymers. The position and degree of *O*-acetylation is genetically determined and varies between organisms, cell types, and developmental stages. Despite the importance of wall polysaccharide *O*-acetylation, only recently progress has been made to elucidate the molecular mechanism of *O*-acetylation. In plants, three protein families are involved in the transfer of the acetyl substituents to the various polysaccharides. In other organisms, this mechanism seems to be conserved, although the number of required components varies. In this review, we provide an update on the latest advances on plant polysaccharide *O*-acetylation and related information from other wall polysaccharide *O*-acetylating organisms such as bacteria and fungi. The biotechnological impact of understanding wall polysaccharide *O*-acetylation ranges from the design of novel drugs against human pathogenic bacteria to the development of improved lignocellulosic feedstocks for biofuel production.

## Plant Cell Wall Polymers are *O*-Acetylated

The biomass of plants contains considerable amounts of esterified acetate. For example, poplar wood contains 5% of its weight as acetate (Johnson et al., 2017), while corn stover contains 4.5% (w/w; Chundawat et al., 2010). Upon processing of the plant biomass the acetate is often released (Selig et al., 2009) not only acidifying the resulting material, but also presenting a potent inhibitor for further downstream microbial fermentation (Helle et al., 2003) such as for the production for biofuels.

The predominant portion of the bound acetate found in plant biomass is present in the cell wall material in the form of *O*-linked acetate on many wall polysaccharides (references below), and to a minor extent on the polyphenol lignin (Ralph, 1996; Del Río et al., 2007). While cellulose, callose, mixed-linkage glucans, and structural glycoproteins are not *O*-acetylated, the dominant matrix polysaccharides including the various pectic polysaccharides and hemicelluloses such as xylan, xyloglucan, and mannans can be *O*-acetylated (Gille and Pauly, 2012). The position and degree of acetylation depends on the wall polymer and can differ not only between plant species, but also cell types, and/or the developmental stage of the plant (Del Río et al., 2007; Obel et al., 2009; Pauly and Keegstra, 2010; Gille and Pauly, 2012; Lourenço et al., 2016). Both the polymer glycan-backbone and/or the side-chain sugar moieties can be *O*-acetylated (Kiefer et al., 1989; Ishii, 1991, 1997; Pauly, 1999; Teleman et al., 2000; Lundqvist et al., 2002; Kabel et al., 2003; O’Neill et al., 2004; Gibeaut et al., 2005; Hoffman et al., 2005; Jia et al., 2005; Hsieh and Harris, 2009; Sengkhamparn et al., 2009). For example, the hemicellulose xyloglucan (XyG) predominantly found in dicot species contains *O*-acetyl moieties on the galactosyl side-chain residues, while in Solanaceous plants and grasses, the glucan-backbone of xyloglucan is *O*-acetylated. In addition, several wall polymers contain sugar-residues that can be mono-/or di-*O*-acetylated (reviewed in Gille and Pauly, 2012).

## Polysaccharide *O*-Acetylation Mechanism

Several lines of evidence suggest that *O*-acetylation of wall polysaccharides takes place as part of the polysaccharide biosynthesis in the Golgi lumen. First, acetylated xyloglucan can be isolated from microsomal preparations suggesting that *O*-acetylation takes place before the wall polysaccharides are secreted into the apoplast (Obel et al., 2009). Second, pectic polysaccharides can be *O*-acetylated *in vitro* in isolated plant microsomes (Pauly and Scheller, 2000). Third, all proteins involved in this modification (see below) are predicted to be located in the Golgi membrane with the putative catalytic domains facing the Golgi lumen (Gille et al., 2011b; Lee et al., 2011; Manabe et al., 2011; Yuan et al., 2013; Schultink et al., 2015; Gao et al., 2017). However, it should be noted that the degree and pattern of polysaccharide *O*-acetylation is also determined by apoplastic plant *O*-acetylesterases, presumably post-deposition in the wall (Gou et al., 2012; Orfila et al., 2012; de Souza et al., 2014; Zhang et al., 2017).

**FIGURE 1:**
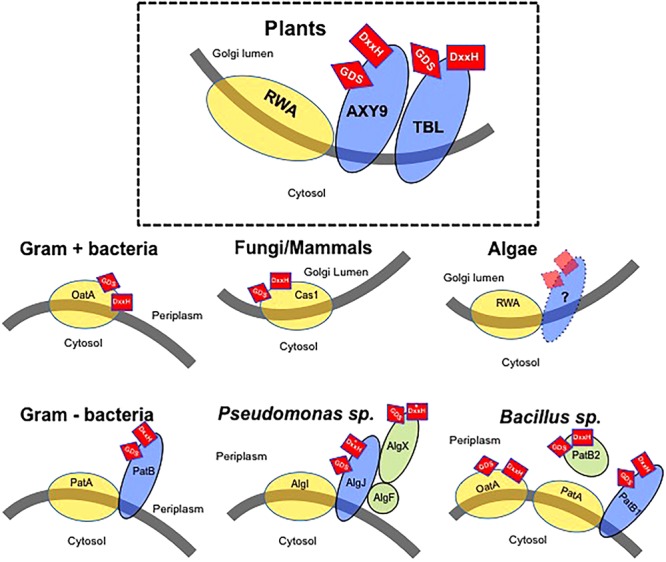
Model of the wall polysaccharides *O*-acetylation mechanism in various organisms. Gray line – Membrane with indication of cellular location of both sides of the membrane. Yellow circles – Proteins consisting of multitransmembrane domains possibly involved in the translocation of an acetyl-moiety. Blue ovals – Membrane anchored proteins with a single-transmembrane domain. Green circles – Soluble proteins associated with *O*-acetylation. Red squares – Presence of GDS and DxxH sequences thought to be required for enzymatic activity. The location of these sequences (side of the membrane) is indicated by their location on the protein(s). Asterisk indicates a variation of the consensus DxxH motif (Baker et al., 2014).

The identification of plant mutants affected in the *O*-acetylation of wall polysaccharides has been instrumental in our understanding of the molecular mechanism of polysaccharide *O*-acetylation. Based on these findings, so far three different protein families are involved in polysaccharide *O*-acetylation (**Figure 1**). One of these protein families is the TRICHOME-BIREFRINGENCE-LIKE (TBL) protein family comprising 46 members in the model species *Arabidopsis thaliana*. Members of the TBL family have been shown to participate in the *O*-acetylation of specific wall polymers. Loss-of-function of the Arabidopsis *ALTERED XYLOGLUCAN 4* (*AXY4/TBL27*) gene results in a complete lack of *O*-acetyl substituents on the hemicellulose XyG without affecting the acetylation status of the other wall polymers (Gille et al., 2011b). Its paralogous gene – *AXY4-like* (*AXY4L/TBL22*) – appears to have the same function but specifically in seeds, indicating that AXY4 and AXY4L are XyG-specific acetyltransferases, although the biochemical activity of both proteins remains to be experimentally demonstrated.

Another well studied example is the Arabidopsis *tbl29/eskimo1* mutant that was shown to reduce xylan *O*-acetylation by 46% in the stem (Xiong et al., 2013). The corresponding TBL29/ESKIMO1 protein was found to catalyze the transfer of *O*-acetyl groups to β-(1→4) xylooligosaccharides *in vitro* thus confirming its role as a xylan *O*-acetyltransferase (Urbanowicz et al., 2014). Recently, the xylan *O*-acetyltransferase activities of other TBL proteins and their regiospecificity of xylose 2-*O*- and/or 3-*O*-acetylation has been demonstrated in Arabidopsis, rice, and poplar (Zhong et al., 2017, 2018a,b). In summary, in Arabidopsis, 9 TBLs lead to xylan 2-*O*-, 3-*O*-monoacetylation or 2,3-di-*O*-acetylation (Zhong et al., 2017). In rice, 66 TBL genes have been identified (Gao et al., 2017). Among these, 14 TBL proteins show xylan 2-*O*- and 3-*O*-acetyltransferase activity (OsXOAT1-14). OsXOAT1-7 are able to complement the defects in xylan *O*-acetylation of the Arabidopsis *esk1/tbl29* mutant (Zhong et al., 2018a). In poplar 64 *TBLs* were identified, 12 of those proteins were shown to *O*-acetylate xylan when heterologously expressed (Zhong et al., 2018b). Other members of the TBL family are thought to be involved in pectin *O*-acetylation such as AtPMR5/AtTBL44, AtTBR, and AtTBL3 (Vogel et al., 2004; Bischoff et al., 2010a) or mannan *O*-acetylation in the case of AtTBL25/AtTBL26 (Gille et al., 2011a). However, in all of these cases enzymatic activity and specificity remains to be demonstrated.

TRICHOME-BIREFRINGENCE-LIKE proteins contain three characteristic protein signatures (**Figure 1**) (Bischoff et al., 2010b). A N-terminus transmembrane domain and two plant-specific domains, DUF231 and TBL. The DUF231 is a domain of unknown function containing a conserved DxxH motif while the TBL motif is characterized by the presence of an esterase GDS motif. The Ser residue from the GDS motif and the Asp and His residues of the DxxH motif are essential for the function of TBL29/ESK1 as mutations of these residues result in a loss of enzyme activity (Zhong et al., 2017).

A second family of proteins involved in polysaccharide *O*-acetylation is represented by ALTERED XYLOGLUCAN 9 (AXY9; **Figure 1**). Arabidopsis mutants affected in *AXY9* expression show a strong reduction in total wall *O*-acetylation in stems and leaf tissues (Schultink et al., 2015). Interestingly, unlike the large, diversified *TBL* gene family, *AXY9* seems to be present in the genome of land plants only as a single copy. Contrary to the polysaccharide substrate specificity of TBL proteins, AXY9 seems to be non-specific in polysaccharide *O*-acetylation as the corresponding *axy9* mutant plants display reductions in *O*-acetylation of multiple hemicelluloses such as xyloglucan or xylan but not pectin. Due to these unique features, AXY9 has been suggested to be involved in the generation of an intermediate acetyl donor substrate used later by TBL proteins (Schultink et al., 2015). The AXY9 protein contains a N-terminus transmembrane domain and a C-terminus facing the Golgi lumen containing GDS and DxxH motifs (**Figure 1**) suggesting that it could also be an *O*-acetyltransferase although if this protein harbors any enzyme activity has yet to be determined.

REDUCED WALL *O*-ACETYLATION (RWA) is the third group of proteins involved in plant polysaccharide *O*-acetylation (Manabe et al., 2011) (**Figure 1**). The Arabidopsis genome contains four RWA proteins required for *O*-acetylation of both pectic and non-pectic polysaccharides including xyloglucan, xylan, and mannan. Quadruple *rwa* mutant plants exhibit a 63% reduction in total wall *O*-acetylation (Manabe et al., 2013). Similarly, down-regulation of the four *RWA* genes found in hybrid aspen (*Populus tremula x tremuloides*) results in reduced wood xylan and xyloglucan *O*-acetylation, suggesting that RWA function is conserved among plant species (Pawar et al., 2017). RWA proteins are characterized by the presence of 10 predicted transmembrane domains (Manabe et al., 2011). In contrast, AXY9 and TBL proteins contain a single transmembrane domain anchoring the protein to the Golgi membrane while the C-terminus of these proteins is oriented toward the Golgi lumen containing the putative catalytic motifs. Despite the lack of amino acid similarity, all these enzymes are predicted to have a short N-terminal cytoplasmic region that has been proposed to act as a signal for retention in the Golgi in the case of other proteins such as glycosyltransferases (Banfield, 2011), although no experimental evidence has been obtained so far for AXY9 or TBL proteins. Also, microsomal preparations isolated from potato cells incubated with radio-labeled acetyl-CoA are able to incorporate and transfer radioactive acetate to proteins and cell wall polysaccharides suggesting that acetyl-CoA is a donor-substrate for the *O*-acetylation of wall polysaccharides (Pauly and Scheller, 2000). As acetyl-CoA cannot diffuse through membranes and the Golgi is not able to produce it (Oliver et al., 2009), it has been proposed that RWA is responsible for the translocation of acetyl-groups across the membrane in order to supply the substrate to the other two families of *O*-acetyltransferases (AXY9 and the various TBLs). Although no experimental evidence has been reported yet, the existence of intermediary acetyl donor(s) is a likely option (Lee et al., 2011; Manabe et al., 2011, 2013; Schultink et al., 2015). In any case, the cytosolic pool of acetyl-CoA is likely the source used by plants for the *O*-acetylation of polysaccharides as it is for alkaloids, anthocyanins, isoprenoids, or phenolics (Fatland et al., 2005; Oliver et al., 2009).

## Similarities with Other Polysaccharide *O*-Acetylating Organisms

All Gram-positive and most Gram-negative bacteria *O*-acetylate extracellular polysaccharides such as their cell wall peptidoglycan (PG) polymer. This heteropolymer is the main component of the bacterial wall, and consists of alternating *N*-acetylglucosaminyl-(β-1,4)-*N*-acetylmuramoyl residues cross-linked with stem peptides. PG *O*-acetylation can occur in 20–70% of the MurNAc residues, depending on the species and growth conditions and provides protection against lytic enzymes such as lysozyme (Moynihan and Clarke, 2011). In the last few years, a great effort has been made to identify and characterize the proteins involved in the *O*-acetylation of PG and other secondary cell wall polysaccharides due to the importance of this modification for the virulence of human pathogens such as *Neisseria gonorrhoeae, Bacillus anthracis*, or *Streptococcus pneumoniae* (Moynihan and Clarke, 2010; Moynihan et al., 2014; Sychantha et al., 2017, 2018). One can find surprising similarities of those systems with the polysaccharide *O*-acetylation mechanisms in plants indicating common ancestry.

In Gram-positive bacteria, OatA proteins consist of a N-terminal RWA-like multitransmembrane domain fused to a globular extracytoplasmic C-terminal domain containing a SGNH/GDSL esterase motif with similarity to plant TBL proteins (**Figure 1**). Hence, Gram-positive bacteria seem to be simultaneously translocating the acetyl groups from a cytoplasmic source and *O*-acetylating the *N*-acetylmuramoyl residues in the extracellular PG polysaccharide using a single bimodular protein. Several OatA homologs have been identified and characterized but only recently the crystal structure of the C-terminal domain of OatA has been resolved and point mutations in the DxxH and GDS motifs demonstrated that these amino acids are essential for catalyzing *O*-acetylation of PG in *Streptococcus pneumoniae* and *Staphylococcus aureus* (Sychantha et al., 2017). A similar protein combination consisting of a globular *O*-acetyltransferase domain combined with multiple transmembrane domains is also observed in fungi and mammals. The fungal CnCas1p protein is responsible for the *O*-acetylation of capsular glucuronoxylomannans in *Cryptococcus neoformans* (Janbon et al., 2001) (**Figure 1**). Although its activity has not been determined experimentally, the human HsCasD1 protein, showing high similarity and structure to CnCas1p, has been demonstrated to be essential and sufficient for *O*-acetylation of sialic acids, a family of nine-carbon monosaccharides typically found capping the glycan chains attached to cell surface glycoproteins and glycolipids in mammals including humans (Arming et al., 2011; Baumann et al., 2015). Similarly to bacterial OatA, activity assays showed that a N-terminus globular domain of HsCasD1 containing the SGNH/GDSL motif catalyzes the 9-*O*-acetylation of sialic acids *in vitro* (Baumann et al., 2015). These results suggest an ancient functional fusion between the multitransmembrane and globular domains in a single protein as a common mechanism to *O*-acetylate extracellular polysaccharides in Gram-positive bacteria, fungi, and mammals (Janbon et al., 2001; Anantharaman and Aravind, 2010; Baumann et al., 2015).

In Gram-negative bacteria multitransmembrane proteins have also been involved in *O*-acetylation of extracellular polysaccharides, such as NolL that *O*-acetylates lipo-chitin oligosaccharides in *Rhizobium* species, or GumG and GumF involved in the acetylation of the mannose residues of xanthan gum produced by *Xanthomonas oryzae* (Pacios Bras et al., 2000; Kim et al., 2009). However, the *O*-acetylation machinery of Gram-negative bacteria consists of multiple proteins as has been observed in plants (**Figure 1**). A multitransmembrane protein might translocate the acetyl moieties from a cytoplasmic source into the periplasm, where one or more plasma membrane-anchored proteins containing a SGNH/GDSL motif facing the periplasm might transfer the acetyl-moiety to the polysaccharide (**Figure 1**). This two-component mechanism involves the coordinated expression of multiple components arranged in operons. A model was originally proposed based on the *O*-acetylation of alginate, a linear exopolysaccharide consisting of 1-4-linked L-mannuronyl and D-glucuronyl residues present in *Pseudomonas aeruginosa* (**Figure 1**) (Clarke et al., 2000). In this bacterial species, the multi transmembrane domain protein AlgI has been suggested to play a similar role as OatA or RWA, exporting the acetyl groups from the cytoplasm. The available acetate would then be used by AlgJ and AlgF proteins, both containing a SGNH/GDSL motif. Although AlgJ and AlgF are both required for alginate *O*-acetylation, their precise functions have not been experimentally demonstrated and it has been proposed that they would not transfer acetyl groups directly to alginate. Instead, they would form a complex that could be acting as an intermediary step providing acetyl groups to AlgX, a protein shown to be able to *O*-acetylate the mannuronyl alginate residues *in vitro* (Baker et al., 2014). According to this model, the intermediate proteins AlgJ and AlgF might be analogous to AXY9 in plants, whereas AlgX would be catalyzing the final step in the *O*-acetylation of alginate, playing a similar role as the TBL protein family in plants. A similar mechanism has been postulated for other Gram-negative bacteria, including *N. gonorrhoeae* or *Campylobacter jejuni* (**Figure 1**) (Weadge et al., 2005; Moynihan and Clarke, 2010; Ha et al., 2016). In these Gram-negative bacteria, several homologs of AlgI (i.e., PatA proteins) are supposed to translocate the acetyl groups through the plasma membrane, whereas PatB proteins catalyze the transfer to the C6 hydroxyl groups of the PG muramoyl residues.

Despite the presence of proteins containing multiple transmembrane domains in both one- and multiple-component polysaccharide *O*-acetylating systems, proteins such as OatA, RWA2 or AlgI share very limited sequence homology with PatA. For example, SaOatA and NgPatA share only 15.1% sequence identity and 23.6% similarity. A similar situation occurs when comparing the *O*-acetyltransferase domain of plant TBL or Gram-positive bacterial OatA proteins with the Gram-negative AlgX or PatB proteins. For example, there is only 15.4% identity and 18.3% similarity between the globular domain of SaOatA and HgPatB (Sychantha et al., 2017). This low degree of sequence similarity suggests different evolutive origins.

Interestingly, some *Bacillus* species seem to have two independent machineries to *O*-acetylate extracellular polysaccharides (**Figure 1**). On the one hand, a bimodal OatA homolog has been characterized exhibiting a mechanism as described above, involving the simultaneous translocation of acetyl groups and PG *O*-acetylation (Laaberki et al., 2011), whereas another system consisting of PatA1 and PatA2 multitransmembrane proteins and the PatB1 periplasmic *O*-acetyltransferase is responsible of the *O*-acetylation of secondary cell wall polysaccharides (Sychantha et al., 2017). Additionally, a second periplasmic protein with demonstrated acetylesterase activity -PatB2- has also been involved in *O*-acetylation of additional cell wall components although the exact donor/acceptor substrate remains to be discovered (Sychantha et al., 2017). Hence, these organisms seem to have developed two different, independent systems for the translocation of acetyl-groups to then specifically *O*-acetylate the various wall polysaccharides utilizing members of two or more *O*-acetyl transferase families.

## Evolution of Plant Polysaccharide *O*-Acetylation Machinery

Gram-positive bacteria, fungi, and mammals developed a one component machinery to *O*-acetylate extracellular polymers. These systems use a single protein combining a multiple transmembrane domain translocating acetyl groups from the cytoplasm fused to a globular domain, containing a SGNH/GDSL-like catalytic motif. In plants, the protein domains and thus functionalities evolved into separate proteins (RWA, AXY9, and TBL protein families, respectively). As plants contain multiple wall polymers an expansion and increased diversification of the TBL protein family might have become necessary. Interestingly, although plant RWA proteins belong to the same sugar acyltransferase superfamily containing 10 transmembrane domains as bacterial OatAs, CnCas1p, and HsCasD1, they lack the globular *O*-acetyltransferase domain, indicating that plants need the additional involvement of other components such as members of the TBL family and/or AXY9 in order to *O*-acetylate their wall polysaccharides. Accordingly, the globular domain of OatA or CnCas1p proteins contains the GDS and DxxH motifs similar to plant TBLs and AXY9 hinting their analogous functions. A similar separate, multiple component mechanism was also developed by Gram-negative bacteria in order to *O*-acetylate extracellular polysaccharides, albeit likely arising through convergent evolution. The development of a multiple component system in these bacteria could reflect again a more complex wall with a variety of extracellular *O*-acetylated polysaccharides. In these bacteria, an increased diversification of the *O*-acetyltransferases is also observed (e.g., PatB1/PatB2 in *B. anthracis* of AlgF, AlgJ and AlgX in *P. aeruginosa*).

**FIGURE 2:**
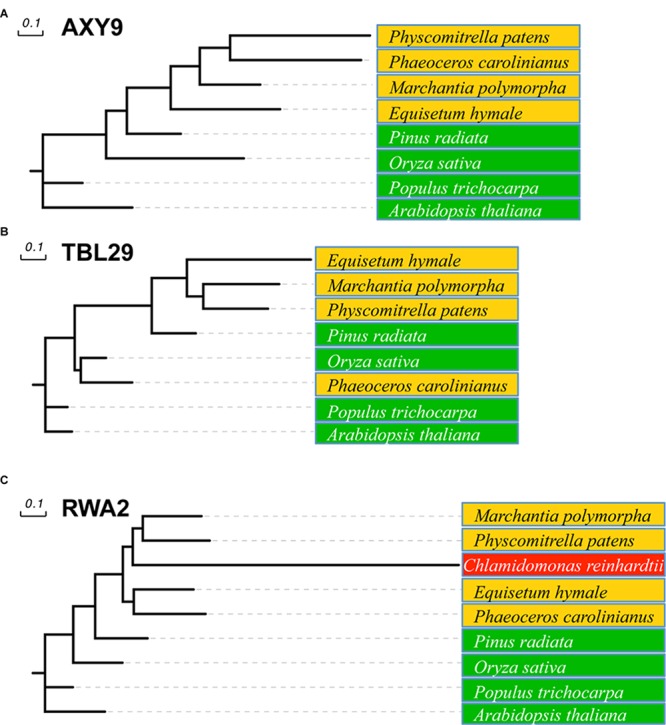
Phylogenetic tree of AXY9, TBL, and RWA proteins. Likelihood tree of AXY9 **(A)**, TBL29 **(B)**, and RWA2 **(C)** protein homologs constructed from sequence alignment of selected species. Green: embryophytes (*Arabidopsis thaliana* and *Populus trichocarpa*, dicots; *Oryza sativa*, monocot; and *Pinus radiata*, gymnosperm). Orange: Bryophyta (*Marchantia polymorpha*, liverwort; *Phaeoceros carolinianus*, hornwort; and *Physcomitrella patens*, moss) and Pteridophyta (*Equisetum hyemale*, horsetail). Red: Algae (*Chlamydomonas reinhardtii*, green algae). Arabidopsis thaliana AXY9, TBL29, and RWA2 protein sequences (UniProtKB references Q9M9N9-1, Q9LY46-1, and Q0WW17-4, respectively) were used in Basic Alignment Search tool protein (BLASTp) against the 1,000 Plants Initiative databases (Matasci et al., 2014; https://db.cngb.org/blast/blastp/) with default parameters and the best hits for every specie were selected for phylogenetic analysis using the Phylogeny.fr web tool with default settings (Dereeper et al., 2008). This tool uses MUSCLE to align the sequences and the Gblocks program to eliminate poorly aligned positions and divergent regions. Phylogenetic trees were then constructed using PhyML using default parameters (Approximate Likelihood-Ratio Test) and the Evolview tool (http://www.evolgenius.info) was used to edit the graphical representation.

All three families of proteins involved in *O*-acetylation of plant wall polysaccharides can be found in vascular plants but also in pteridophytes and bryophytes, including hornworts, mosses, and liverworts (**Figure 2**). A sequence comparison of nine representative embryophytic species showed that AXY9, TBL29, and RWA2 proteins seems to be highly conserved in dicots (*Arabidopsis thaliana* and *Populus trichocarpa*), monocots (*Oryza sativa*) and gymnosperms (*Pinus radiata*) sharing identities higher than 50% and similarities around 75% with the *Arabidopsis* representatives. Primitive plants such as *Equisetum hyemale*, liverworts (*Marchantia polymorpha*), hornworts (*Phaeoceros carolinianus*) and mosses (*Physcomitrella patens*) also contain highly conserved sequences sharing identity and similarity values around 40% and 60%, respectively.

Land plants evolved from Charophyte green algae after their separation from Chlorophyte green algae (Lewis and McCourt, 2004; Becker and Marin, 2009). Although during the transition from an aquatic to terrestrial environment cell walls in both algae and plants have evolved independently (Niklas, 2004), it is still likely that some of the wall components have a common ancestry (Popper and Tuohy, 2010). Accordingly, the biosynthetic machinery of some of the polysaccharides present in a typical plant wall (e.g., xylan) can be traced back to the Charophyte green algae (Jensen et al., 2018). When probing algal genomes with the Arabidopsis RWA2 sequence, homologs can be found in dozens of green algae species including members of both the Chlorophyta (e.g., *Volvox aureus* or *Nephroselmis pyriformis)* and Charophyta (e.g., *Klebsormidium subtile* or *Coleochaete scutata*) divisions. However, algae seem not to encode proteins with sequence similarity to AXY9 or TBL29. Since algal RWA orthologs do not contain a GxxH and/or GSD domain required for polysaccharide *O*-acetylation algae might harbor additional, hitherto unidentified proteins that would be necessary for *O*-acetylation to occur. These results indicate that RWA proteins emerged earlier than AXY9 and the TBLs and suggest that green algae may also use a polysaccharide *O*-acetylation system based on RWA. The walls of several Chlorophyta and Charophyta species have been reported to contain plant-type wall polysaccharides such as xylan, mannans or XyG (Painter, 1983; Lahaye et al., 1994; Lahaye and Robic, 2007; Popper et al., 2011). Unfortunately, information about the *O*-acetylation status of these organisms is missing probably due to the alkali-based methods used during wall isolation.

## Biological Significance of Polysaccharide *O*-Acetylation

*O*-acetylation of polysaccharides, including the various hemicelluloses and the pectic polysaccharides homogalacturonan and rhamnogalacturonan I, influences the polymer’s physiochemical properties. Addition of *O*-acetyl-moieties contribute to the gelling properties and viscosity of the isolated polymers an important issue for food applications (Rombouts and Thibault, 1986; Huang et al., 2002). *O*-acetyl substituents increase polysaccharide hydrophobicity and lead to conformational changes that influences interactions with other polymers, either supporting binding or due to steric hinderance abolish interaction (Busse-Wicher et al., 2014). As a result, de-*O*-acetylation through, e.g., alkali-treatments leads often to a decrease in solubility in aqueous environments and precipitation of polymers (Gibeaut et al., 2005; Busse-Wicher et al., 2016). Moreover, enzymatic attack of the polymer by glycosyl hydrolases is restricted due to steric hindrance in the vicinity of the target glycosidic bond (reviewed by Biely et al., 2016). As an application example in the wood industry, biomass chemical treatments include chaotropic alkali and acetic anhydride treatments in order to de-acetylate and re-acetylate the lignocellulosic polysaccharides, respectively, to modify the wood properties. De-acetylation improves properties for pulping, saccharification and fermentation due to the properties mentioned above. On the other hand, chemical acetylation of wood increases mechanical strength, durability and resistance to fungi, bacteria, and termites, as acetylation of xylan and mannan increases the stiffness and allows interactions with hydrophobic substances such as lignin (reviewed in Pawar et al., 2013). However, in non-lignified tissues, de-acetylation of primary wall polysaccharides (e.g., pectic polysaccharides) has been associated with increased cell wall stiffness probably due to a close spatial association between pectin and cellulose microfibrils (Gou et al., 2012; Orfila et al., 2012).

*In planta* the biological significance of a particular polysaccharide *O*-acetylation pattern is diverse and in many cases not clear. For example, a complete lack of XyG sidechain *O*-acetylation has no apparent impact on plant growth and development as the wild-type (WT)-like phenotypes of Arabidopsis *axy4* and *axy4L* knockout mutants demonstrate. Reinforcing this notion, a natural *Ty-0* Arabidopsis accession displays an almost complete lack of XyG *O*-acetylation without detrimental plant morphological and developmental side-effects when grown in its native environment in the Highlands of Scotland (Gille et al., 2011b). However, *O*-acetylation seems to affect the aluminum binding capacity of XyG as demonstrated by an increased aluminum content in the hemicellulose fraction in *axy4* mutant roots compared to the WT when growing in the presence of this metal (Zhu et al., 2014). Yet, one cannot rule out the possibility that XyG *O*-acetylation may play a role in other environmental adaptation processes including specific stresses and/or growing conditions yet to be identified. In contrast to dicots such as Arabidopsis or poplar, in the grasses and plant members of the Solanaceae (such as tomato, tobacco, etc.) the glucan-backbone of XyG is partially *O*-acetylated (Gibeaut et al., 2005; Jia et al., 2005). This is caused by XyG *O*-acetyltransferases such as the Brachypodium *BdXyBAT1* that *O*-acetylate the non-xylosylated glucosyl backbone residues (Jia et al., 2005; Liu et al., 2016). When *BdXyBAT1* is expressed in Arabidopsis, the backbone of XyG becomes *O*-acetylated reducing the degree of xylosylation of XyG indicating that *O*-acetylation impacts negatively the addition of other substitutions (Liu et al., 2016). A reduction of the size of glycosyl side-chains of XyG lacking, e.g., the fucosyl and galactosyl residues leads to retarded plant growth (Pauly et al., 2013; Schultink et al., 2013). It is thought that this dwarfism is caused by a distorted matrix polysaccharide secretion system caused by the poor solubility of the less substituted XyG (Jensen et al., 2012; Kong et al., 2015). However, the addition of backbone *O*-acetyl substituents to this lowly substituted XyG in the Arabidopsis mutant results in a reversion of the retarded growth (Liu et al., 2016). These results indicate that *O*-acetylation of the XyG glucan-backbone is functionally equivalent to glycosyl-sidechains and might represent an energetically favorable strategy by replacing C5 and C6 carbon sugars with C2 acetates (Gibeaut et al., 2005; Jia et al., 2005; Gille and Pauly, 2012; Liu et al., 2016).

Mutants affected in xylan *O*-acetylation display multiple pleiotropic phenotypes including dwarfism, altered plant architecture and constitutive stress-related responses associated with a vascular collapse. Xylan is a major component of the walls present in the water conducting xylem. Hypoacetylation of xylan seems to affect the physical strength of the xylem walls, as they are not able to resist the negative water pressure generated during water transport. As a consequence, mutants affected in members of the AXY9, RWA or particular TBLs that impact xylan *O*-acetylation all display alterations in plant growth and development. In Arabidopsis, the *axy9-2* mutant shows an 80% reduction in xylan *O*-acetylation and a strong growth arrest (Schultink et al., 2015), whereas the quadruple *rwa* mutant shows a 42% reduction in xylan *O*-acetylation with a reduction in secondary wall thickening and collapsed xylem morphology (Lee et al., 2011). Regarding the TBL family, only *tbl29/esk1* single mutant alleles, with a 40% reduction in xylan *O*-acetylation, show a clear irregular xylem phenotype. Several other *tbl* single mutants with only minor reductions in xylan *O*-acetylation show only additive effects in the corresponding double, triple or multiple mutant combinations in vascular development and plant growth in several plant species (Yuan et al., 2016a,b,c; Gao et al., 2017).

In addition to the xylem collapse and growth arrest, xylan hypoacetylation has also been associated with other developmental and stress-related phenotypes. *tbl29/esk1* mutant alleles also show stress-related pleiotropic phenotypes such as increased tolerance to drought, salt or freezing, likely an indirect consequence of the collapsed xylem (Xin and Browse, 1998; Xin et al., 2007; Bouchabke-Coussa et al., 2008; Lefebvre et al., 2011; Ramirez et al., 2018). Intriguingly, several lines of evidence seem to indicate that low xylan acetylation may not be directly responsible for these observed phenotypes. For example, expression of fungal acetyl-esterases in *Arabidopsis* and *Brachypodium* causes post-synthetic de-acetylation of xylan but it does not impact plant development or xylem morphology (Pogorelko et al., 2013b). Most recently, the identification of two *tbl29* suppressors, where the xylem collapse and growth arrest are recovered but the wall/xylan acetate remains reduced, strongly supports these observations. *KAKTUS* (*KAK*) loss of function increases stem diameter and activates the development of larger tracheary elements. As a consequence, *kak* mutations are able to recover almost completely from *tbl29/esk1*-associated dwarfism without affecting wall acetate content. Although KAK has been described previously as an endoreduplication repressor affecting trichome morphology, the mechanism how it regulates vascular development is not known (Downes et al., 2003; El Refy et al., 2003; Bensussan et al., 2015). Altered biosynthesis and/or perception of some plant hormones (e.g., abscisic acid; ABA) have been suggested to play a role in the pleiotropic phenotype of *tbl29/esk1*. *tbl29/esk1* alleles show increased ABA levels and enhanced expression of several ABA-dependent genes, but genetic evidence discarded that this hormonal pathway is directly responsible for the phenotypes of *tbl29/esk1* plants. Double mutants blocking ABA biosynthesis or perception in a *tbl29/esk1* background are not able to recover the developmental defects shown by the *tbl29/esk1* single mutant. Moreover, increased ABA perception and *tbl29/esk1* down-regulation seems to have additive effects on drought tolerance, suggesting that they affect independent pathways (Lefebvre et al., 2011). These findings indicate that altered ABA levels are more a consequence of the pleiotropic phenotype of the *tbl29/esk1* mutant rather than the cause. In contrast, it has been shown that blocking strigolactone (SL) synthesis in *tbl29/esk1* plants (i.e., *tbl29 max4* double mutants) is able to completely suppress both developmental defects and increased freezing tolerance without affecting the reduced acetate content (Ramirez et al., 2018). In addition, exogenous applications of a synthetic SL to *tbl29 max4* plants result in dwarfism and collapsed xylem, further confirming that these phenotypes are SL-dependent. This suggests that an altered SL pathway could be directly involved in leading to the pleiotropic phenotypes associated to the *tbl29/esk1* mutants. As SLs are hormones involved in the regulation of multiple plant processes including stem elongation, secondary growth, leaf expansion and adaptation to abiotic stress (reviewed in Waters et al., 2017), this opens the possibility that xylan hypoacetylation could be perceived by an unknown mechanism triggering the activation a SL-dependent response regulating xylem development (Ramirez et al., 2018).

In addition to acetate, xylan can also be substituted with (methyl-)glucuronic acid (methyl-GlcA) residues by xylan glucuronosyltransferases termed GUX. Together, these decorations have been found to be important for xylan-cellulose binding (Mortimer et al., 2010; Rennie et al., 2012; Bromley et al., 2013; Busse-Wicher et al., 2014). Actually, vascular plants seem to generate a specific xylan decoration pattern as acetate and GlcA are found spaced on even-numbered residues in the xylan backbone (Busse-Wicher et al., 2016). Recently, it has been shown that in *Arabidopsis*, TBL29/ESK1-dependent xylan *O*-acetylation is required for the generation of the even-patterned GlcA substitutions (Grantham et al., 2017). In a *tbl29/esk1* mutant, where xylan acetylation is reduced, GUX1 is unable to maintain the GlcA decoration pattern suggesting that a correct *O*-acetylation pattern is required for the addition of GlcA residues. As a consequence of this uneven substitution, xylan might not be able to acquire the typical twofold screw ribbon conformation impeding its docking onto the hydrophilic face of a cellulose microfibril to form semicrystalline xylanocellulose fibrils (Grantham et al., 2017). Intriguingly, expression of GUX1 in vascular tissue under the control of a tissue specific promoter is able to rescue the *tbl29/esk1* mutant growth defects indicating that xylan functionality is restored (Xiong et al., 2015). GUX1 is able to glucuronosylate additional available positions on the xylan backbone due to the absence of *O*-acetyl-groups in *tbl29*. Glucuronosylation of xylan can thus be considered functionally equivalent to *O*-acetylation *in vivo*. This agrees with the notion that the addition of *O*-acetyl substituents (C2 units) to wall polysaccharides instead of sugars (C5-C6) could have evolved as a more energetically favorable strategy as described above for XyG. Other TBL proteins than TBL29/ESK1 participate in the regiospecific *O*-acetylation of xylan (Zhong et al., 2017), suggesting the existence of a precisely regulated mechanism to create a tissue-specific *O*-acetylation pattern in xylan in order to adequately interact with cellulose and likely other cell wall components.

*O*-acetylation of pectic polysaccharides has also been associated with plant signaling processes. *rwa2* mutant alleles show increased resistance to the necrotrophic fungal pathogen *Botrytis cinerea* accompanied by leaf surface defects including trichome collapse, enhanced leave permeability and altered cuticle formation (Manabe et al., 2011; Nafisi et al., 2015). As these defects have not been observed in other mutants affected in hemicellulosic polysaccharide *O*-acetylation, it has been speculated that these phenotypes may be caused by pectin hypoacetylation although direct evidence is still lacking (Nafisi et al., 2015). Other reports have associated reduced pectin *O*-acetylation with increased disease resistance. For example, plants overexpressing a fungal rhamnogalacturonan acetylesterase constitutively activate defense responses and show increased resistance to pathogens (Pogorelko et al., 2013b). Since a similar response has been observed after application of oligogalacturonide fragments (OGs) and more efficiently by partially acetylated OGs, it has been proposed that pectin *O*-acetylation might be involved in a cell wall integrity maintenance system (Randoux et al., 2010; Pogorelko et al., 2013a).

Recently, pectin *O*-acetylation has been also proposed to regulate other important developmental processes such as photomorphogenesis (Sinclair et al., 2017). Both the *tbr* mutant, affected in a putative pectin acetyltransferase, and the *rwa2* mutant, show a photomorphogenic response when grown in the dark. This phenotype can be restored by adding small homogalacturonan fragments and thus pectin *O*-acetylation might regulate a dark signal involved in a complex network of light-dependent seedling development (Sinclair et al., 2017).

## Open Questions

There are still open questions regarding the mechanism of polysaccharide *O*-acetylation not only in plants but also in bacteria, fungi, and mammals. First, the identity of the acetyl-donor and the detailed mechanism of translocation through the Golgi membrane are not known in any of these organisms. Although it is likely that the cytosolic acetyl-CoA pool is tapped for this purpose, acetyl-CoA itself is likely not transferred. Studying this process is challenging as the likely responsible protein contains multiple transmembrane domains. Second, the exact mechanism of the transfer of an acetyl group from a donor to the hydroxyl group of an acceptor sugar remains unknown, although mechanistic insights became recently available from bacterial OatA proteins (Sychantha and Clarke, 2018). Recent reports in bacteria have suggested a direct acylation of the OatA protein following a ping-pong bi-bi mechanism of action where the acetyl group is covalently attached to the catalytic Ser residue of the enzyme before being transferred to the substrate. A similar acetyl-enzyme intermediate has also been proposed in the Gram-negative PatA/PatB mode of action, where PatB *O*-acetyltransferases could form a complex with the acetyl-bound PatA membrane proteins precluding free water from accessing the active site, preventing the hydrolysis of the translocated acetyl group ensuring an efficient acetate transfer (Moynihan and Clarke, 2010). The existence of likely intermediary steps as suggested in other Gram-negative bacteria systems (e.g., AlgI/AlgF/AlgJ/AlgX) could implicate the formation of a multiprotein complex for the *O*-acetylation of extracellular polysaccharides. A RWA/AXY9/TBL complex formation could also be conserved in plant systems, although mechanistic details are still missing. Comparison of the various polysaccharide *O*-acetylation systems raises the question of the evolution of the various *O*-acetylation mechanism – in essence why multiple proteins are required for this process in some species while in other species apparently a single protein suffices. Third, the transferases responsible for the *O*-acetylation of some wall polymers (e.g., mannans, pectins, or lignin) remain to be discovered, albeit it is likely that members of the TBL family are involved. The identification and characterization of such proteins is not only needed to understand the wall *O*-acetylation mechanism of particular wall polysaccharides, but also to gain insights into the function of the *O*-acetyl substituent on this polymer. Fourth, the recent advent of identifying the genes responsible for polysaccharide *O*-acetylation and their genetic manipulation *in vivo* lead to the discovery of intriguing function of this substituent. However, at this stage the phenotypic results are rather descriptive and additional research in the future is required to ascertain causal relationships as well as mechanistic insights into polymer interactions, cellular sensing and responses.

## Author Contributions

MP and VR designed and wrote the manuscript.

## Conflict of Interest Statement

The authors declare that the research was conducted in the absence of any commercial or financial relationships that could be construed as a potential conflict of interest.
